# Availability of Plastic and Reconstructive Services (PRS) for Head and Neck Reconstruction with Free Flaps (FF) Following Oncological Resection in India—A Reality Check

**DOI:** 10.1007/s13193-025-02317-5

**Published:** 2025-05-06

**Authors:** Shivakumar Thiagarajan, Agrim Jain, Akansha Kondoi, Dushyant Jaiswal, Vinaykant Shankdhar, Gouri Pantvaidya

**Affiliations:** 1 Division of Head & Neck, Dept of Surgical Oncology, Homi Bhabha National Institute (HBNI), Tata Memorial Centre, Mumbai, India; 2 Homi Bhabha National Institute (HBNI), Tata Memorial Centre, Mumbai, India; 3 Department of Plastic & Reconstructive Surgery, Homi Bhabha National Institute (HBNI), Tata Memorial Centre, Mumbai, India

**Keywords:** Oncoreconstruction, Head and neck, Plastic and reconstructive services, India

## Abstract

Head and neck cancer is among the most common cancers in India. Most of these patients present with advanced disease requiring extensive surgical resection and appropriate reconstruction. Though the expertise for surgical resection may be available, the same for reconstruction, especially microvascular reconstruction, may not always be available. We included centres that were members of National Cancer Grid (NCG) and institutes that offered academic programs such as the M.Ch, DNB (Surgical Oncology and Head and Neck Surgery), FNB (Head and Neck Oncology), and Fellowships (Head and Neck) across the country. After identifying the centres, we analysed how many of these centres had a department of Plastic and Reconstructive Surgery (PRS) and whether they offer reconstructive services, including microvascular free flaps. Three hundred and sixty-eight centres were identified across India. One hundred and eighty-eight centres (46%) had a PRS department, most of them were in the south zone (*n* = 57/90, 63.3%) and north zone (*n* = 33/65, 60%) (*p* < 0.001), in private centres (*n* = 135, 58.9%) (*p* < 0.001), in tier 1 cities (*n* = 58/100, 58%) (*p* < 0.001), and centres with active academic programs (*p* < 0.001). Out of 188 centres, 166 performed microvascular free flaps (MFF). In a few centres, reconstruction was done by surgeons who performed the resection of the cancer (*n* = 54, 14.6%). Overall, MFF was performed in 57.2% of hospitals across the country. There is a reasonable number of centres with PRS services available in the country for oncoreconstruction. However, their distribution seems to be skewed, with more of them located in private institutions/centres and tier 1 cities.

## Introduction

**Fig. 1:**
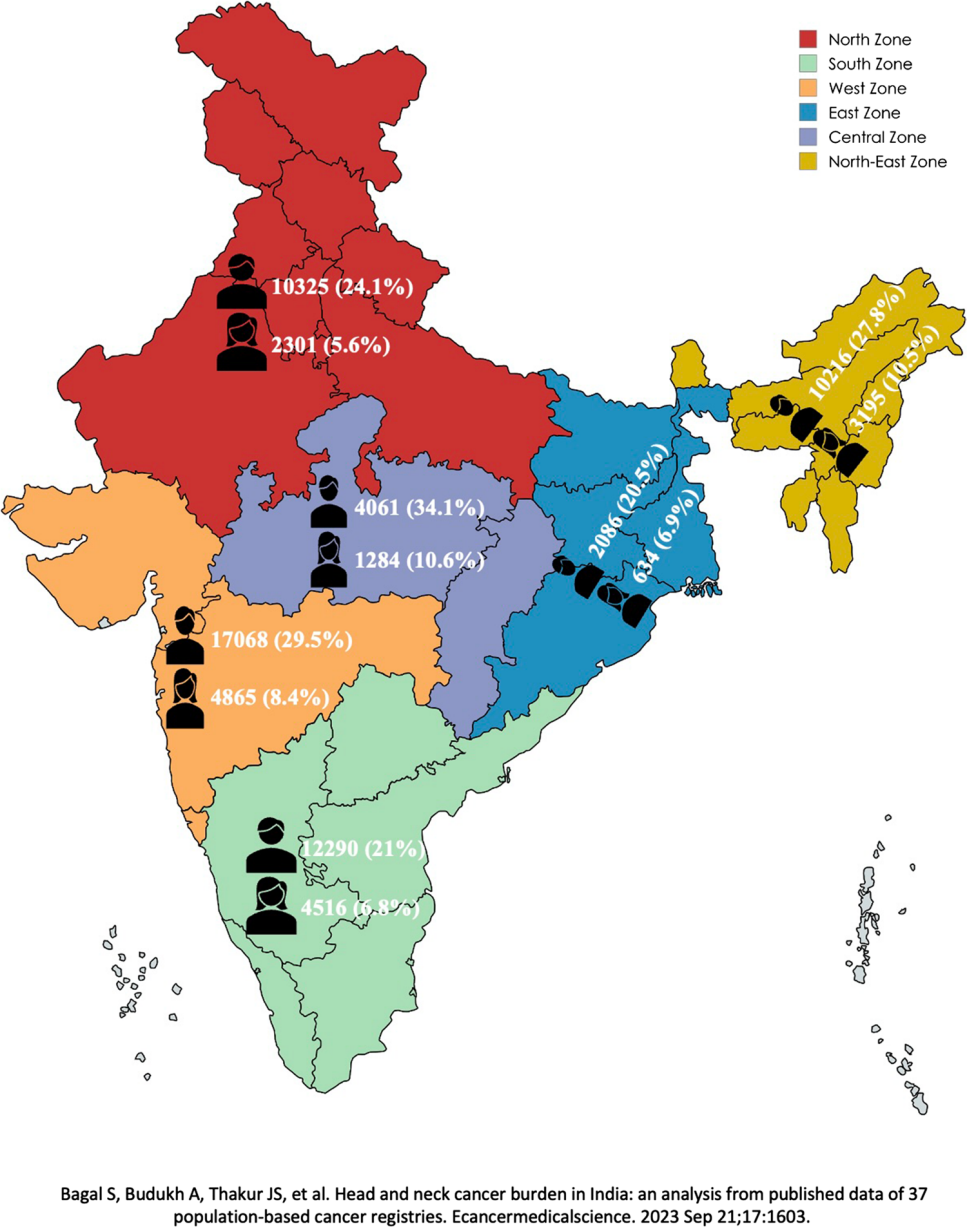
Depicts the head and neck cancer burden in males and females in India

Oral cancer ranks as the 16 th most common cancer globally, with approximately 389,846 new cases and around 200,000 deaths reported annually [1]. India bears a significant portion of this burden, contributing to nearly one-third of the global cases (Fig. 1) [2]. Bagal S and colleagues have mentioned in their study that 1 in every 33 males and 1 in every 107 females are at risk of developing head and neck cancer. They have also noted that mouth is the leading cancer site [2]. In India, a significant challenge is the late diagnosis of cancer, with around 66.6% of cancer patients diagnosed with locally advanced disease, necessitating major resections and complex reconstructive surgeries [3]. Reconstruction options include pedicled and free flaps (FFs) following resections for these advanced cancers. Over the past 20 years, reconstruction with FF has been considered the standard of care, as it contributes to improved oncologic outcomes, better function, and lower morbidity [4].

The clustering of Plastic and Reconstructive Surgery (PRS) experts, notably those with FF experience, within prominent medical facilities in urban areas, poses a significant obstacle to delivering optimal cancer care, particularly in remote/rural regions. In a survey spanning 16 cancer centres across India, the average annual usage of pedicle flaps/locoregional flaps (PF/LF) accounted for 64.5%, while FF comprised 35.5%. This was attributed to various reasons, including the availability of expertise [5]. Acquiring empirical data regarding the availability of PRS services, especially to carry out FF, is vital to comprehensively understanding the prevailing availability of the expertise in the country and identifying gaps, if any, to bring out the requirements for the policymakers to act upon. Such insights are pivotal for implementing targeted interventions to ensure equitable access to appropriate reconstruction, especially FF, throughout India. Hence, in this article, we aimed to understand the pattern of availability of PRS with expertise in FF across centres offering cancer treatment in India.

## Methodology (Fig. 2)

**Fig. 2:**
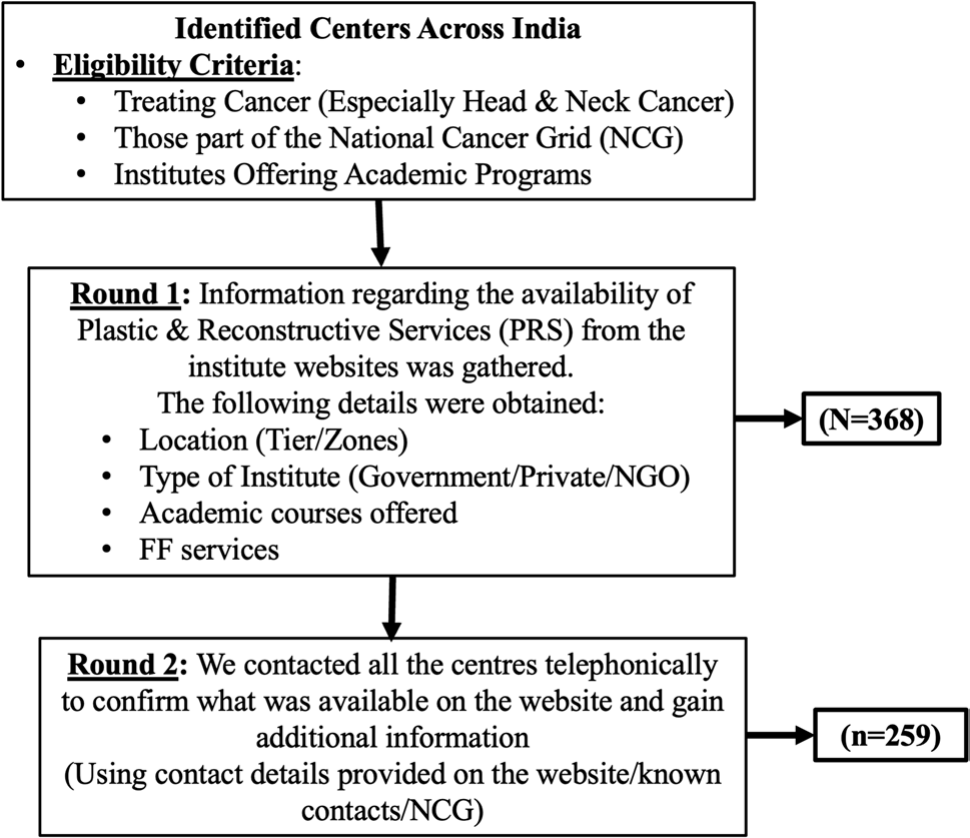
Study methodology

We performed this study in line with the Declaration of Helsinki, which was revised in 2013. The primary objective was to understand the pattern of availability of PRS across centres in India offering cancer treatment. The secondary objective was to identify gaps/disparities, if any. We included centres treating cancer (Especially Head and Neck Cancer), those part of the National Cancer Grid (NCG) and institutes Offering Academic Programs. We accessed all the resources available in the public domain and identified the state and regional cancer centres across India, centres within the National Cancer Grid (NCG) network and institutes offering academic programs such as M.Ch, DNB (Surgical Oncology and Head and Neck Surgery), FNB (Head and Neck Oncology) and Fellowships (Head and Neck) nationwide. We meticulously screened through this comprehensive list and visited the individual websites of these centres/institutions, and the information regarding the availability of PRS, especially for head and neck cancer patients, was obtained. In round 1, we attempted to obtain information about the geographical location of these centres and their funding structure, i.e. government-funded, private or non-governmental organisations (NGOs), availability of in-house academic programmes and availability of FF services from the website. Subsequently, in round 2, we attempted to contact these centres through the available contact details and/or through known contacts from these centres to confirm the information that was available on the website. In addition, we asked the following questions to all centres that we contacted, i.e. do you have a PRS department at your centre (Yes/No); do you have a facility for in-house FF services at your centre (Yes/No); if not do you call plastic and reconstructive surgeons from outside for this (Yes/No); how many FF are done (approximate numbers) annually at your centre; if not for plastic and reconstructive surgeons who else does FF at your centre.

Statistical analysis was done using SPSS version 29 (IBM Corp, Armonk, NY). Descriptive analyses were done. Also, the *χ*^2^ test was done to understand the significance of the association between various factors regarding the availability of the PRS and the performance of FF across centres. A *p* value of < 0.05 was considered significant.

## Results

Three hundred and sixty-eight centres were identified across India, satisfying the eligibility criteria (Fig. 2). We could gather all the information mentioned in the flowchart (Fig. 2) from the provided websites in round 1 for the respective institutes/centres. Subsequently, in round 2, when we contacted these centres to obtain further information and confirm the information on the webpage, 259 centres (70.3%) responded to our calls. We could not reach out to the remaining centres as the contact details provided were inaccurate. Overall, out of the 368 centres, 19 were designated as Regional Cancer Centres (RCC), 11 as State Cancer Institutes (SCI), and 16 as Tertiary Cancer Care Centres (TCCC). Figure 2 gives an overall perspective of PRS/FF services availability across India.

### PRS Departments (Table 1)

**Table 1:** Plastic and Reconstructive Surgery (PRS) departments are distributed across the tiers of cities and zones, as well as the nature of institutes and academic courses offered at their respective institutes

Variables	PRS department: yes	PRS department: no	*p* Value
Tier of the city			
Tier 1 Tier 2 Tier 3	58 (58%) 90 (49.7%) 26 (32.5%)	42 (42%) 91 (50.3%) 54 (67.5%)	0.003
Zones			
North South Central East West Northeast	33 (60%) 57 (63.3%) 12 (35.3%) 12 (38.7%) 58 (44.9%) 2 (14.2%)	22 (40%) 33 (36.7%) 22 (64.7%) 19 (61.3%) 81 (55.1%) 12 (85.8%)	< 0.001
Institute type			
Government Private NGO/Trust	32 (30.5%) 135 (58.9%) 7 (28%)	73 (69.5%) 94 (41.1%) 18 (72%)	< 0.001
Academic centres			
Yes No	109 (52.9%) 65 (41.9%)	97 (47.1%) 90 (58.1%)	0.03

**Fig. 3:**
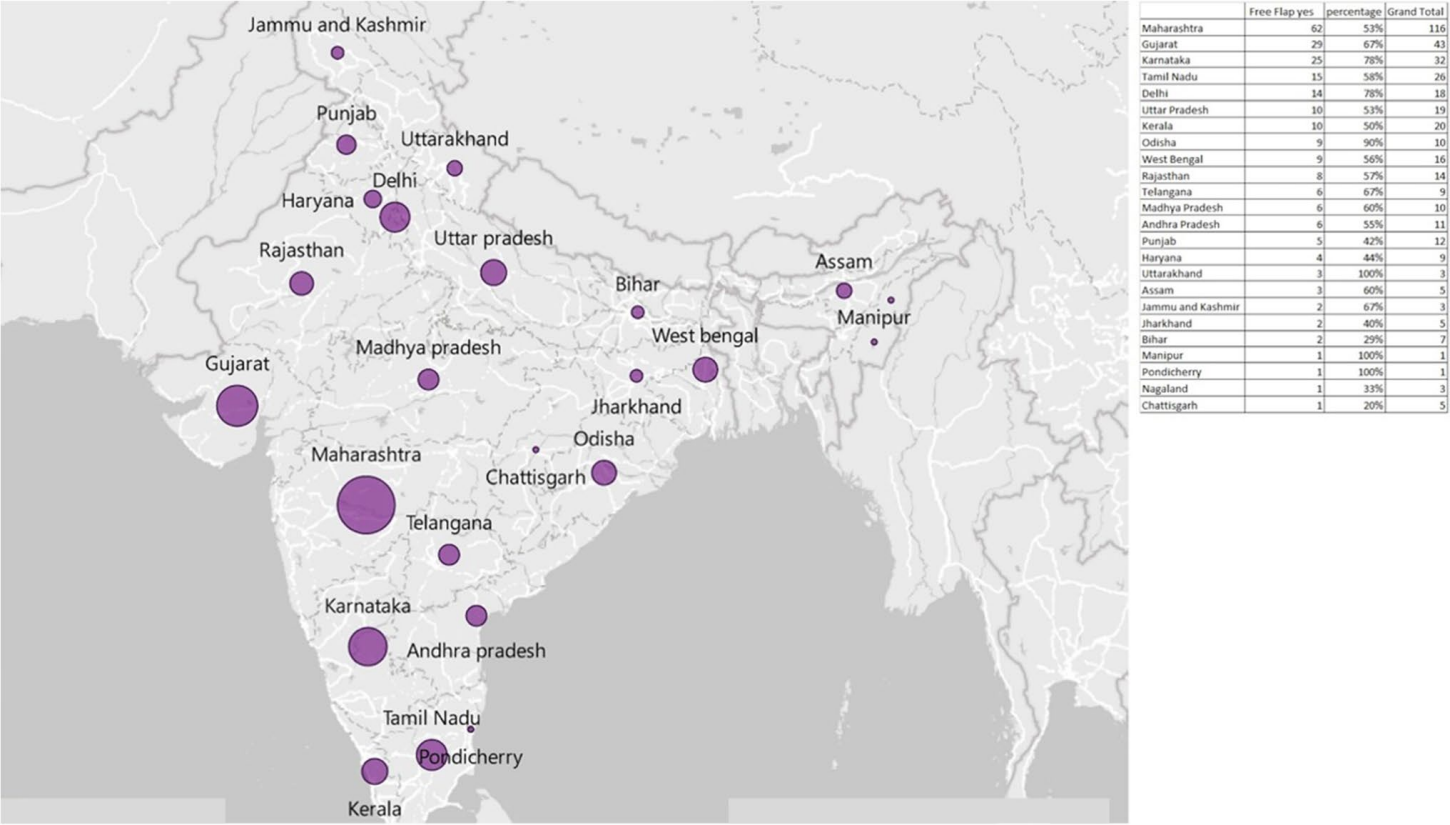
Heat map showing the state-wise numbers of centres treating cancer (grand total) and the number of centres within them performing free flaps

Of the 368 centres, 188 (46%) had a dedicated PRS department, indicating that a significant proportion of centres offering cancer treatment in India provide these services. Very few RCCs (36.8%, 7/19), SCIs (36.36, 4/11) and TCCCs (25%, 4/16) had dedicated PRS departments. Geographically, most identified centres (where cancer surgeries, especially Head and Neck, were performed) were in Maharashtra 116 (28.4%) (Fig. 3). The number of PRS departments were available most often in tier 1 cities (*n* = 58/100, 58%) compared to tier 2 (*n* = 90/181, 49.7%) and tier 3 (*n* = 26/80, 32.5%) cities (*p* = 0.003). Forty-one Plastic and Reconstructive Surgeons were offering their services in different hospitals/nursing homes in tier 1 cities on an on-call basis. The distribution of PRS departments and microvascular FF services across various zones in India highlights significant regional variations (*p* < 0.001). The south zones (*n* = 57/90, 63.3%) and north zone (*n* = 33/65, 60%) had the most PRS departments compared to west zone (*n* = 58/139, 44.2%), east zone (*n* = 12/31, 38.7%), central zone (*n* = 12/34, 35.3%) and northeast zone (*n* = 2/14, 14.2%). Across the country, the private centres (135/229, 58.9%) were most likely to have both PRS departments and FF services compared to government centres (*n* = 32/105, 30.4%) and centres run by NGOs (*n* = 7/25, 28%). The presence of PRS services was more commonly seen in academic centres than non-academic centres.

###   Reconstruction with Microvascular FFs

**Fig. 4:**
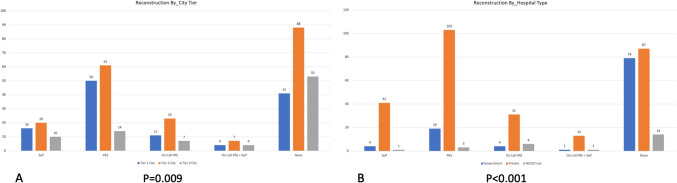
Depicts who is performing reconstruction across tiers of cities (**A**) and hospital type (**B**)

FF services were offered following resections by 166 centres with PRS departments. In the remaining 22 centres, no FF was being done despite the presence of a PRS department (*p* < 0.001). A good percentage of reconstruction is being done by the surgeons performing the resection of the cancer (*n* = 54, 14.6%). In 15 centres, the PRS surgeons were performing reconstruction (microvascular free flap) on a need basis in addition to the surgeons performing the resection of cancer who were also performing free flaps. Overall, FF was performed in 57.2% of hospitals across the country. Around 117 centres, when contacted telephonically, could give us the approximate number of free flaps being done per year at their centres. Most of the centres (*n* = 73) performed 10–100 free flaps in a year. Some high-volume centres were performing 100–500 free flaps per year (*n* = 51). Most of these high-volume FF reconstructions were being performed in private institutions in tier 1 cities (Fig. 4B). High-volume FF reconstructions were being done in some government institutions such as ours. Table 2 gives us a glimpse of the FF services available zone-wise, as per tier of city and academic vs non-academic centres. The FF being done in the northeast zone was the least compared to the other zones in the country. In tier 2 cities, the FF reconstruction was done mostly by the same surgeon who performed the ablative surgery (resection) (Fig. 4A). The majority of the PRS surgeons who were not attached to any institute (freelancing) performed 100–200 FF per year.

**Table 2:** Distribution of free flaps being performed across the tier of cities, zones, nature of institute and academic courses offered at their respective institutes

Variables	Free flaps: yes	Free flaps: no	*p* Value
Tier of the city			
Tier 1 Tier 2 Tier 3	84 (68.8%) 114 (57.2%) 36 (40.9%)	38 (31.2%) 85 (42.8%) 52 (59.1%)	< 0.001
Zones			
North South Central East West Northeast	34 (58.6%) 62 (62.6%) 19 (52.7%) 23 (58.9%) 92 (56.4%) 4 (40%)	24 (41.4%) 37 (37.4%) 17 (47.3%) 16 (41.1%) 71 (43.6%) 10 (60%)	0.278
Institute type			
Government Private NGO/Trust	30 (28%) 193 (70.1%) 11 (44%)	77 (72%) 82 (29.9%) 14 (66%)	< 0.001
Academic centres			
Yes No	115 (55.8%) 78 (50.3%)	91 (44.2%) 77 (49.7%)	0.299

## Discussion

Our study, for the first time, provides an insight into the landscape of availability of PRS services, especially with FF expertise across cancer centres offering treatment for head and neck cancers. As per the findings, PRS services are available in 46% of the centres offering surgery for cancer, especially head and neck cancer in India. A good percentage of reconstruction is being done by the surgeons performing the resection of the cancer (*n* = 54, 14.6%). The free flap services are offered mostly in private centres and tier 1 cities. Also, centres that were performing more than 100 free flaps per year were often situated in tier 1 cities (*p* = 0.007). Most of the RCCs, SCIs and TCCCs do not have PRS services or FF in their centres. This is concerning and alarming and needs immediate attention. The absence of it is reflected in the low percentage of FF in these centres. The availability of FF in the private sector, trumping government/institutional setups, points to a need for institutional upgrades for microvascular setups. Also, the reconstructive services available in the northeast are less and require attention.

The presence of 41 on-call/freelance Plastic and Reconstructive surgeons performing FF ranging from 100 to 300 a year is a significant observation. It points to a skill-driven rather than a setup-driven practice, FF often being done by a single surgeon in a small setup. It might be the road ahead for a large resource-constrained nation with disparity/deficiency in tier 2/3 and smaller places. It also debunks the myth of requiring institutional setups and large teams for microsurgery.

Head and neck cancer is among the common cancers in India, and most of these patients present with advanced-stage disease [1, 2]. Surgery forms an important part of the treatment for patients with head and neck cancers, which would necessitate appropriate reconstruction to restore form and function [3]. Decision-making and flap selection of reconstruction options following resection have evolved from the traditional reconstructive ladder to the elevator approach to the toolbox and supermarket approaches [6]. For this, the uniform availability of expertise across the country with the various reconstruction options suitable for the defect created is essential [5]. Reasonable evidence to substantiate this was not available till now, though this was perceived by all caregivers [5]. Hence, in this article, we aimed to provide this crucial information to the policymakers to close the gap that may exist.

Vaidya et al. [5], in their article, mention that the utilisation of FF varied from 1.5 to 88.9%, though the cumulative usage was 35.5% for FF. They noticed that centres with a greater proportion of self-paying capacity/with private insurance received FF more often. At the same time, those availing of public health insurance schemes facilities had more pedicled or local flap utilisation. Most regional cancer centres with a high volume of work used FF on fewer occasions. Some of the major roadblocks to doing FF regularly were longer waiting lists and lesser compensation under the public health insurance schemes.

In our study, we found that FF services are not offered in all PRS departments (*n* = 22, 11.7%). In India, close to 250 seats (Both M.Ch and DNB) are available every year for training in PRS across the country [4]. Exposure and training in FF during their specialisation both in PRS and Head and Neck Surgery may be helpful.

Maharashtra seems to be an outlier in terms of PRS and FF availability in absolute numbers and percentages, with deep penetration not only in tier 1 but also in tier 2 and 3 places. This is even in comparison with other states of similar economic and development profiles. This could be due to the presence of multiple PRS academic degree programs in the state, which provides the manpower.

Some suggestions that could help improve the present scenario are as follows: firstly, to identify centres of excellence for teaching and training in FF following cancer surgery in every zone of India. Second, ensure the presence of a PRS/FF capable team/unit (full-time or on-call) with every Head and Neck cancer team/unit. Third, an institutional mandate for a full-time PRS unit/team with all RCC, SCI, and TCCC, which could again be on-call/part-time only if logistically feasible. Fourth, the availability of an operating microscope in all Head and Neck Cancer Surgery units. Fifth, to take necessary steps to improve the government health scheme packages for FF, maybe have a separate add-on for FF. Also, consider the possibility of incentivising FF reconstruction for tier 2 and 3 cities. Lastly, mandate basic microvascular training for all PRS/Head and Neck Surgery trainees as part of the training.

Our article is among the first to give a more wholesome insight into the availability of PRS and FF facilities/services across the country, especially among centres treating cancers, particularly head and neck cancer. It also provides information essential for stakeholders and policymakers to act upon and improve.

## Conclusions

Reconstructive services are available in up to 46% of centres that treat head and neck cancers in India. Free flaps were being done in 57.2% of hospitals overall. These numbers, though more than perceived, are far from ideal. Most of these services are available in private centres and tier 1 cities. These services are available in centres with some form of actively running academic programmes. The findings from this study would help policymakers address the disparities identified.

## Data Availability

Data can be made available on reasonable request.
